# The migration and fusion events related to ROCK activity strongly influence the morphology of chicken embryo intestinal organoids

**DOI:** 10.1007/s00709-018-1312-3

**Published:** 2018-10-16

**Authors:** Małgorzata Pierzchalska, Małgorzata Panek, Maja Grabacka

**Affiliations:** Department of Food Biotechnology, Faculty of Food Technology, The University of Agriculture in Kraków, Balicka 122, 30-149 Kraków, Poland

**Keywords:** Intestinal organoids, Collective cell movement, Organoid fusion and rotation, Rho-associated protein kinase, Lipopolysaccharide, Chicken

## Abstract

The method of organoid culture has become a tool widely used in gastrointestinal research, but so far, the migration of organoids derived from gut epithelium and formed in 3D Matrigel matrix has not been reported and studied. The intestinal epithelial tissue derived from 19-day-old chicken embryo was cultured in Matrigel and the dynamic properties of the forming organoids were analyzed by time-lapse image analysis. It was observed that about one in ten organoids actively moved through the matrix, at a speed of 10–20 μm/h. Moreover, rotation was observed in the majority of organoids that did not migrate long distances. The fusion events took place between organoids, which collided during the movement or growth. In our previous paper, we showed that the presence of Toll-like receptor 4 ligand, *Escherichia coli* lipopolysaccharide (LPS, 1 μg/ml), increased the mean organoid diameter. Here, we confirm this result and demonstrate that the Rho-associated protein kinase (ROCK) inhibitor Y-27632 (10 μM) did not completely abolish organoid migration, but prevented the fusion events, in both LPS-treated and untreated cultures. In consequence, in the presence of Y-27632, the differences between cultures incubated with and without LPS were not visible. We conclude that migration and fusion of organoids may influence their morphology and suggest that these phenomena should be taken into account during the design of experimental settings.

**Electronic supplementary material:**

The online version of this article (10.1007/s00709-018-1312-3) contains supplementary material, which is available to authorized users.

## Introduction

The collective cell migration is of profound importance for development, tissue morphogenesis, regeneration, and cancer progression in multicellular organisms, but its mechanism is not fully understood, because this phenomenon is difficult both to observe in vivo and to analyze in vitro (Scarpa and Mayor 2016)*.* The normal epithelial tissue used to be considered as relatively static. Recently, however, it has been suggested that epithelial sheets may transit from a jammed (more static) to an unjammed (more fluidic) state (Pegoraro et al. 2016). It was also previously reported that epithelial spheroids of vertebrates formed in 3D conditions (Rørth 2012) and *Drosophila* organ-like structures called egg chambers rotate in an actin and myosin-dependent manner (Viktorinová et al. 2017) therefore exhibiting a sort of collective cell migration.

In the last few years, a diversity of organoid cultures has been successfully developed, leading to numerous publications based on the technique (Simian and Bissell 2017). The methods of spheroid and organoid cultures turned out to be particularly important for gastrointestinal studies. Since the pioneering papers, published almost 10 years ago, describing the mouse enteroids derived from intestinal crypts or stem cells embedded in the Matrigel layer (Sato et al. 2009; Ootani et al. 2009), various gastrointestinal organoids have become a popular tool in research into gut physiology. Although these new methods certainly hold great promise, they also raise some important concerns. The standardization and interpretation of results in organoid research could be difficult due to structural and histological complexity, as well as dynamic changes in morphology during the time of culture (Zachos et al. 2016).

Many papers published recently have analyzed changes in the number, size, and shape of the intestinal organoids cultured under various conditions. The results are usually interpreted in the context of the balance between stem cell proliferation and differentiation. In such papers, the area or diameter and the number of organoids per well (i.e., seeding efficiency) are commonly calculated parameters (Beyaz et al. 2016, Matsumoto et al. 2016). We have recently established the method of culture of chicken intestinal organoids comprising both epithelial and mesenchymal cells (Pierzchalska et al. 2012) and demonstrated that their growth is boosted by the presence of some Toll-like receptor (TLR) ligands or probiotic bacteria in the culture milieu. Organoids cultured in the presence of live *Lactobacillus acidophilus* LA5, LPS from *Escherichia coli* O111:B4 (TLR4 agonist) or Pam3CysSerLys4 (TLR2 agonist) were significantly larger compared with organoids cultured in standard conditions (Pierzchalska et al. 2017).

Here, we focus on image analysis revealing the remarkable dynamic and migratory properties of chicken intestinal organoids and suggest that the generally overlooked collective cell movement and fusion events may profoundly affect the morphology of the organoids.

## Materials and methods

### Organoid culture

The embryonated eggs from Ross 308 hens were obtained from a local professional hatchery (Krak-Drób, Sciejowice, Poland). Embryonic chicken intestinal epithelium fragments were isolated from small intestines removed from decapitated 19-day-old embryos, as previously described (Pierzchalska et al. 2016). The medium composition was as follows: DMEM/F12 (PAA Laboratories GmbH, Pasching, Austria) with antibiotic-antimycotic solution (Zell Shield; Minerva Biolabs, Berlin, Germany), insulin-transferrin-selenium premix (BD Biosciensces, San Diego, CA, USA), and epidermal growth factor (EGF, 25 ng/mL), R-spondin 1 (500 ng/ml), Noggin (25 ng/ml), and WNT3a (10 ng/ml) (all R&D Systems, Minneapolis MN, USA) with 5 μg/mL PGE_2_ (Cayman Chemical, Ann Arbor, MI, USA) and chicken serum (0.25%, PAN Biotech, Aidenbach, Germany). The cell culture inserts located in the compatible 12-well culture plates (0.4-μm pore size, transparent; BD Biosciences) were coated with 200 μl Matrigel™ (Matrigel Basement Membrane Matrix, Corning, USA) tissue suspension 1 to 1 mixture. In some experiments, ultrapure LPS from *Escherichia coli* O111:B4 (1 μg/ml, InvivoGen, France) or Rho protein kinase inhibitor, Y-27632 (10 μM, Seleckchem, Munich, Germany) were added to the medium at the point of establishing the organoid culture. The medium (750 μl) was transferred to the lower chamber and, after jellification of the Matrigel layer, to the upper chamber (250 μl) of insert-containing wells.

### Time-lapse microscopic image analysis and calculation of parameters defining organoid culture appearance

The cultures were observed with an inverted Axio Observer Z1 microscope equipped with a thermostatic incubation chamber with CO_2_ influx (Zeiss, Munich, Germany). For time-lapse recording, the images from at least five fields of view from each culture were captured with an AxioCam HR digital camera. The recording was controlled by Zen 2012 software and images were collected at 30-min intervals using a × 5 or × 10 objective. The recording started about 1 h after the establishment of the culture and lasted for 48–72 h. The organoid projection borders were contoured manually using the Zen 2012 graphical tools, in each field of view (at least 50 organoids from one culture) and the mean areas of organoid projection were calculated, as well as the mean number of organoids in one field of view (the mean ± SD of at least five measurements are presented on graphs). The fusion events were counted in the first 2 days of culture by the analysis of time-lapse films (five films per experimental point). Statistical analysis was performed using Statistica 9 software. An ANOVA test confirmed the statistical significance of the differences in parameter characteristic of the cultures kept in the presence or absence of Y27632 (the *p* value below 0.05 was considered significant).

### Total cellular protein isolation and immunoblotting

The Matrigel matrix containing organoids was detached from insert membranes, washed twice with cold PBS, and incubated in Matrisperse (BD Biosciences) on ice for 30 min. The released organoids and cells were centrifuged (1000×*g* for 5 min.) and the pellets were lysed with RIPA buffer (Thermo Fisher Scientific) Then, 20 μg of total cellular protein were subjected to PAGE in 8% polyacrylamide gels and transferred to the nitrocellulose membrane. Protein extracts were also prepared with a RIPA buffer from tissue fragments of chicken intestinal epithelium after centrifugation of the suspension (1000×*g*, 4 °C). The chicken embryonic intestines were incubated in chelating agent as with the establishment of the culture. The immunodetection was undertaken as previously described (Panek et al. 2018) using the following antibodies: polyclonal anti-sucrase-isomaltase (1:200, SAB2102141, Sigma-Aldrich, St. Louis, MO, USA), monoclonal anti-α-smooth muscle actin (1:1000, 1A4, Sigma-Aldrich, St. Louis, MO, USA), monoclonal antibody against chicken villin (1:2000, MCA292, AbD Serotec, Raleigh, NC, USA). Immunodetection of reference protein was performed on the same membrane without stripping, using the anti-growth factor receptor-bound protein 2 mouse monoclonal antibody (BD Transduction laboratories) diluted 1:3000. The signal was visualized using SignalFire™ ECL Plus Reagent (Cell Signaling Technology Inc., USA) and X-ray films.

## Results

**Fig. 1:**
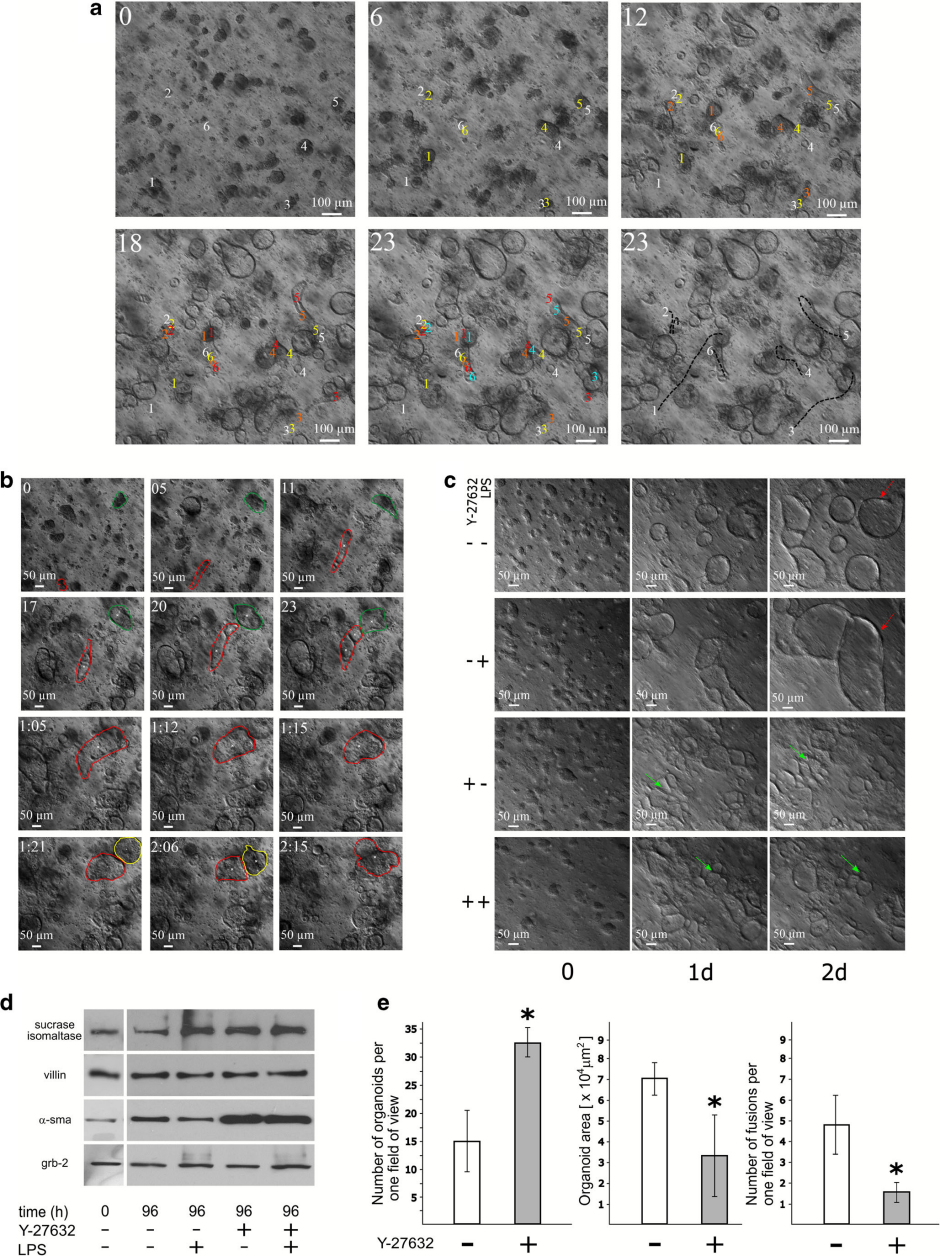
The dynamic of organoids growth—the migration and fusion events in various culture conditions. The formation of organoids was recorded under an inverted microscope equipped with an incubation chamber, by collecting one image every half an hour in the first 3 days of culture. **a** The location of organoids moving through the Matrigel parallel to the insert surface was marked by numbers (1–6); the position of given organoid center at time 0 is shown with a white number; and at 6 h—yellow, 12 h—orange, 18 h—red, and 23 h—blue. The black dashed lines on the photograph in the lower right corner of the panel represent the distances covered by moving organoids in the first whole day of culture (see also Suppl. video 1). **b** The organoids moving towards one another and the fusion events between them were also recorded. The boundaries of three individual organoids that fused into one structure were contoured in red, green, and yellow. The lumen formation is marked by a white cross (see also Suppl. video 2). **c** The presence of ultrapure lipopolysaccharides from *Escherichia coli* 0111:B4 (LPS, 1 μg/ml) in the medium caused the formation of larger organoids on days 2 and 3 from seeding, as compared to the organoids cultured without such stimulation (C, red arrows). The addition of Y-27632 (10 μM) resulted in the formation of more organoids but of smaller size, regardless of the LPS presence (C, green arrows, see also Suppl. video 4). **d** The epithelial lining obtained by incubation of the intestines of 19-day-old chicken embryos in PBS contacting EGTA and glucose for 2 h was used for total protein isolation and subsequent immunoblotting (time 0). The tissue fragments isolated in the same way were used to start the organoid cultures. Organoids were cultured for 3 days without LPS and Y-27632 or with LPS (1 μg/ml) or with Y-27632 (10 μM) or both. Upon completion of the time-lapse video recording, the media were exchanged and cultures were transferred to the incubator. After 96 h of culture, the total cellular proteins were isolated and the presence of enterocyte markers (sucrase-isomaltase and villin), myofibroblasts marker (α-smooth muscle actin—α-sma), and loading control (growth factor receptor-bound protein 2—grb2) was detected by immunoblotting. The protein samples from organoid cultures treated with Y-27632 inhibitor contain much more α-sma. **e** The projection area of organoids and number of organoids in one field of view were calculated from digitized images of cultures kept with the absence (open bars) or presence (closed bars) of ROCK inhibitor, which were taken at 72 h from seeding. All fusion events occurring in the first 48 h were calculated by a time-lapse video analysis (the mean of five independent measurements is presented). An ANOVA test was performed to confirm the significance of the difference between organoids cultured with and without Y-27632: *p* value below 0.05 was considered significant and marked by asterisks (see also Suppl. video 4)

The epithelial fragments isolated by the chelation of calcium ions vary in shape and size. The analysis of total proteins derived from initial intestinal tissue preparation by immunoblotting revealed the presence of villin (which is a protein marker of both mature enterocytes and their progenitors), sucrase-isomaltase (a differentiated enterocytes marker), and a very small amount of α-sma (a marker of myofibroblasts) (Fig. 1d). In approximately 1 h, simultaneously with the jellification of the surrounding Matrigel, some fragments of epithelium closed up, forming structures which were more compact and had a diameter of between 10 and 40 μm. Observation of the growth of organoids in the first 24 h revealed that the majority of those fragments enlarged rapidly, reaching about 200 μm in diameter, and simultaneously transformed into hollow spheres covered with epithelial cells accompanied by a few myofibroblasts (organoids with empty lumens are formed between 4 and 10 h). Time-lapse recording indicated that a substantial number of tissue fragments not only grew into organoids but also moved inside the Matrigel matrix. The migratory activity observed in about 10% of organoids started as early as the first 2 h of culture and was independent of lumen formation. The moving organoids reshaped much more rapidly and frequently had an elongated morphology. Typically, the more static an organoid, the more spherical was its morphology (Fig. 1a, Suppl. video 1). The distances covered by organoids in 24 h varied greatly (mean value 292, range 144–458 μm). In consequence of the growth and movement, the separate tissue fragments bumped into each other in Matrigel and eventually fused. The frequency of fusion events was high and the majority of organoids on the second day of culture were engaged in at least one fusion event (Fig. 1b, Suppl. videos 1 and 2). Over time, fusion events and migration became occasional whereas the rotation remained the most frequently observed type of movement. Nevertheless, some organoids with empty lumen in the 2-day-old cultures could still move and fuse (Suppl figure. 1, Suppl. video 3).

We previously showed that the addition of lipopolysaccharide (LPS) can influence the growth of organoids during the first 3 days of culture in Matrigel, by triggering the formation of larger structures with more irregular shapes as compared to organoids not treated with this TLR4 agonist (Pierzchalska et al. 2017). Here, we used a ROCK inhibitor (Y-27632, 10 μM), both alone and simultaneously with LPS, to check whether the inhibitor was able to reverse the LPS action. We observed the increase in spheroid numbers and the decrease in their size, both in LPS-treated and untreated cultures. The reason for this could be the lower rate of organoid fusion in inhibitor-treated culture (Fig. 1e). The analysis of total cellular protein isolated from 4-day-old cultures indicates that stimulation with LPS resulted in a slightly increased expression of a marker of differentiated enterocytes and sucrase-isomaltase. Treatment with Y27632 inhibitor did not abolish this effect. On the contrary, even ROCK inhibition by Y-27632 alone caused an increase in the marker expression of enterocytes. Moreover, the stimulation of mesenchymal cell growth by ROCK inhibition is suggested by the fact that a myofibroblast marker, α-SMA, is more abundant in the samples from inhibitor-treated cultures (Fig. 1d). The presence of numerous small organoids strongly attached to each other was the most striking morphological feature of cultures treated with Y-27632 (Fig. 1c). The analysis of organoid behavior on time-lapse recorded films indicates that although the inhibitor is not able to abolish migration of spheroids, it acts powerfully in preventing the fusion events (Fig. 1e, Suppl. video 4).

## Discussion

The time-lapse video recording of the chicken embryo intestinal organoid cultures in Matrigel clearly demonstrates that intestinal organoids cannot only fuse, as others incidentally reported in mammalian systems (Jabaji et al. 2014, Sachs et al. 2017), but also simultaneously show migratory activity—rotation and directional movement. Such observation prompts immediate questions as to why the phenomenon was mostly overlooked by others and what is the mechanism responsible for such dynamic events.

Despite some excellent recent studies on the cohesive movement of various epithelial cell clusters through dense 3D extracellular matrix (Sharma et al. 2015, Sato et al. 2015, Hiraiwa et al. 2017), the understanding of this process remains elusive. It is generally supposed that such movement must engage the mechanical forces related to the surrounding matrix remodeling (Gjorevski et al. 2015). In this connection, it is important to emphasize that the methodology we used for culture establishment is slightly different to that generally applied for mammalian organoids. We prepared the mixture of Matrigel and intestinal tissue fragments in a suspension in which the final concentration of Matrigel reached 50%. A drop (200 μl) of this mixture was distributed onto the surface of porous membranes of cell culture inserts. We found this procedure to be an effective way of supporting the growth of the organoids, as compared to the method in which the cells are embedded in more concentrated Matrigel directly covering the cell culture dish. It emerged that organoid movement was also promoted by the environment with lower stiffness (data not shown).

It is also worth noticing that—in contrast to mammalian systems—chicken embryo intestinal organoid culture comprises myofibroblasts. It seems that fibroblasts are important in the collective movement of tumor cells in 3D, enhancing their invasiveness. For example, it was shown that migration of squamous cell carcinoma cell clusters into the surrounding matrix is led by fibroblasts (Gaggioli et al. 2007) and collective cellular movement of ameloblastoma cells was enhanced in vitro by the presence of stromal fibroblasts (Fuchigami et al. 2017). It is possible that in the case of chicken intestinal organoids, the presence of myofibroblasts boosts effective organoid migration. As far as the cooperation of various cell types inside organoids is concerned, one can take into account the reorganization of adherens junctions and the force of transmission through them. It was demonstrated that remodeling of adherens junctions controls cell-cell cooperation during collective cell migration of MDCKII monolayers by the α-catenin-related mechanism (Matsuzawa et al. 2018) and some studies point to the specific role of various cadherins, e.g., E-cadherin and P-cadherin, in collective cell movement in 3D (Plutoni et al. 2016). Similarly, atypical Fat2 cadherin of *Drosophila melanogaster* is supposed to regulate collective cell movement during tissue rotation (Squarr et al. 2016). Although such detailed analysis was beyond the scope of the current communication, it would be interesting in the future to investigate the reorganization of adherens junctions and the role of their constituent proteins in moving intestinal organoids.

The inhibition of Rho-associated coiled coil-containing protein kinases (ROCKs) activity was shown to prevent some types of individual cell migration, as of *Amoeba proteus* (Kłopocka and Redowicz 2004) or breast cancer cells (Guerra et al. 2017) and also some types of collective cell movement (Mikami et al. 2015). However, it was also reported that ROCK inhibitors enhance other migratory activities, such as the movement of various cell types: endothelial cells from spheroids (Breyer et al. 2012), human proximal tubular epithelial cells, and epithelial cells of intestinal origin (Hopkins et al. 2007). The ROCK inhibitor Y27632 is also widely used as a stimulant of stem cell survival, which increases the efficiency of the formation of intestinal organoids (Han et al. 2017).

The ROCK activity is generally considered profibrotic, promoting the transition of epithelial-mesenchymal and fibroblasts-myofibroblasts (Ji et al. 2014). On the other hand, some authors concluded that the Y-27632 inhibitor augments myofibroblast proliferation and migration in culture (Piltti et al. 2015). It was also demonstrated that ROCK inhibitors primed human-induced pluripotent stem cells to selectively differentiate towards mesendodermal lineage (Maldonado et al. 2016), which is in line with our observation of the increased proportion of myofibroblasts in 4-day-old organoid cultures treated with the ROCK inhibitor. Though it is not clear whether the abundance of myofibroblasts plays any role in counteracting fusion events, one can speculate that their localization in close proximity to the epithelial walls of organoids may preclude the contacts between the sheets of epithelial cells of the congested spheres.

Two recently published studies dealt with dynamic behavior of gastrointestinal organoids: one demonstrated that human gastric epithelial spheroids could rupture, rotate, and fuse (Sebrell et al. 2018) and the other showed some contractile activity of neonatal murine intestinal organoids (containing mesenchymal cells) cultured in an air-liquid interface (di Marco et al. 2014). So far, the migration of enteroids or organoids through the Matrigel matrix has not been studied and we are convinced that the mechanism of this sort of collective cell movement deserves future attention. Obviously, further systematic research is required to find out if the migratory properties of organoids described in this report are characteristic for fetal tissue only, or if the elastic properties of the matrix (e.g., Matrigel concentration) can influence the shape changes and movement of organoids.

When organoids are being used as model systems mimicking the intestinal tissue (so-called mini guts models) to test the influence of various compounds or microorganisms on mucosal physiology, the comparison of such culture parameters as the diameter and number of organoids is routinely performed. In this sort of experiment, fusion events and possible migratory activity of organoids are generally overlooked, especially if time-lapse video recording is not performed. This might lead the authors of such studies to conclude that the larger the organoid diameter, the more stem cells proliferate in the organoid wall, but that might not always be the only explanation. When organoids are microinjected with harmful pathogens (as, e.g., in Dutta and Clevers 2017), commensal bacteria (as, e.g., Karve et al. 2017), or with beneficial or toxic food components, to mimic their presence in the intestinal lumen, the fusion between organoids might also be a cause of misinterpretation of the experimental results. The same problems could occur when inside-out and outside-in transports between a lumen and an extracellular space or the process of lumen acidification are studied. Therefore, we conclude that migration and fusion events should not be neglected during the design of experimental settings, when the impact of various factors on the morphology of organoids is analyzed.

## Electronic supplementary material

Supplementary figure 1 **The dynamic properties (rotation and migration) of chicken embryo intestinal organoids cultured in Matrigel matrix.** The movement of organoids was recorded between day 2 and day 3.5 of culture. It is worth noticing that even organoids with similar morphology located in close proximity can behave differently – one remains in place and rotates (black arrowheads show a direction of rotation), while another actively changes its shape, moves and fuses with another organoid (red arrow, see also Suppl. video 3). (PNG 3.47 mb)

Supplementary video 1 **The formation and dynamic properties of chicken embryo intestinal organoids cultured in Matrigel matrix.** The organoid cultures were observed by time-lapse video microscopy and images were captured at two frames per hour over a period of 68 h. The video was generated from these and plays at 7 frames per second. (AVI 74902 kb)

Supplementary video 2 **The formation and dynamic properties of chicken embryo intestinal organoids cultured in Matrigel matrix.** The organoids were observed by time-lapse video microscopy and images were captured at two frames per hour over a period of 68 h. The video was generated from these and plays at 7 frames per second. (AVI 88057 kb)

Supplementary video 3 **Rotational movement observed in chicken embryo intestinal organoids cultured in Matrigel matrix.** The organoids were observed by time-lapse video microscopy and images were captured at two frames per hour between 24 and 48 h of culture. The video was generated from these and plays at 7 frames per second. (AVI 38561 kb)

Supplementary video 4 **The comparison between the dynamic properties of organoids cultured in Matrigel matrix in the presence or absence of LPS or ROCK inhibitor.** The panel contains four typical movies generated from the time-lapse video recording of 4 independent cultures from the same experiment. Organoids were treated with ultrapure lipopolysaccharides from *Escherichia coli* 0111:B4 (1 μg/ml, LPS), ROCK inhibitor, Y-27632 (10 μM, ROCK inh.), LPS and Y-27632 (LPS and ROCK inh.), or kept without presence of these factors (Con). The LPS stimulation caused the formation of larger organoids as compared to control. Regardless of LPS presence, the addition of Y-27632 resulted in formation of higher number of organoids but with smaller size. (AVI 69975 kb)
