# Supplementary figures and images for: The migration and fusion events related to ROCK activity strongly influence the morphology of chicken embryo intestinal organoids
Source: Protoplasma. 2018 Oct 16;256(2):575–81. doi: 10.1007/s00709-018-1312-3 (PMC6514079; doi:10.1007/s00709-018-1312-3)

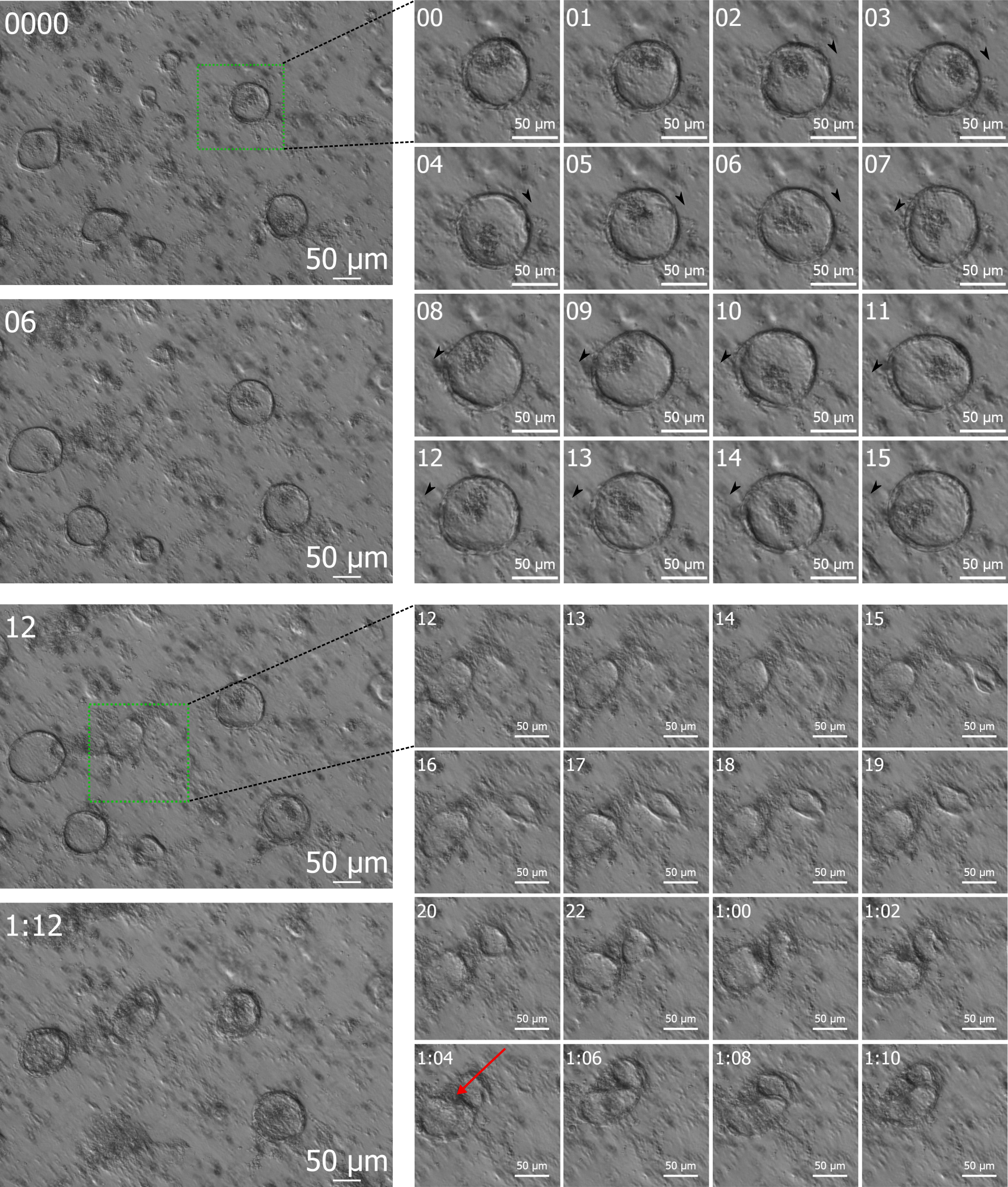

Supplement: Supplementary file 1 — The dynamic properties (rotation and migration) of chicken embryo intestinal organoids cultured in Matrigel matrix. The movement of organoids was recorded between day 2 and day 3.5 of culture. It is worth noticing that even organoids with similar morphology located in close proximity can behave differently – one remains in place and rotates (black arrowheads show a direction of rotation), while another actively changes its shape, moves and fuses with another organoid (red arrow, see also Suppl. video 3). (PNG 3.47 mb) [file 709_2018_1312_MOESM1_ESM.png]
